# New Appliances and Things Medical

**Published:** 1907-07-27

**Authors:** 


### New Appliances and Things Medical.

[We shall be glad to receive at our Office, 28 & 29 Southampton Street, Strand, London, W.C., from the manufacturers, specimens of all new preparations and appliances which may be brought out from time to time.]

### TOKAY WINE.

(Messrs. Berry, Bros. and Co., 3 St. James's Street, London, S.W.)

We have to thank Messrs. Berry, Bros. and Co. for sending us samples of this most interesting, and, at any rate in this country, not very well known wine. The name "Tokay" is given to a wine which is produced from a special kind of grape which grows in a district of Hungary called Hegyallya, which, as a matter of fact, is a high-lying region between Tokay and Satorallya. For the making of Tokay the grapes are allowed to become so ripe that they shrivel, they are then carefully collected, subjected only to a very slight kneading, and the juice which flows out of them allowed to ferment. This is the actual process followed in the making of the so-called essences, but in the case of the more ordinary wines (Ausbruch) the dried grapes are pressed in the ordinary way and are mixed with wine made from fresh grapes of the same sort, that is, before they have shrivelled or become partly converted into raisins. The characteristic of Tokay is that it is a wine of relatively low alcoholic strength but containing a very high extract, or, in other words, a very full body. The alcoholic strength of Tokay rarely exceeds 14, and is often as low as 9 and 10 per cent. Some Tokay wines contain sugar, others are practically free from it. With regard to its body we must look upon Tokay as comparable to Port or Madeira; but from the alcoholic standpoint it is much lighter than either of these wines, and is certainly in many cases much better borne. We think Tokay is a wine that in this country has been neglected by the medical profession to the disadvantage of their patients; it certainly possesses many advantages from the dietetic standpoint, and where a light but distinctly tonic and restorative wine is indicated may prove to be exceedingly useful. The samples which we have received from Messrs. Berry Bros. are of good quality and pleasing flavour and bouquet.

### NOLET'S AROMATIC SCHNAPPS.

(Agents: Messrs. Bulfield and Co., 37 Crutched Friars, E.C.)

We have received two sample bottles of Messrs. Nolet's Schiedam Aromatic Schnapps. The preparation before us is a spirit of good quality to which certain aromatic substances are added, chief among these being the active principle of the juniper berry. Oil of juniper has unquestionably marked diuretic properties after absorption into the blood, and before absorption exerts in the stomach an antiseptic and a carminative action. This cordial is therefore not only useful when a urinary stimulant is required, but also in cases of fermentative dyspepsia associated with—as they most often are—a sluggishly acting gastric muscle. It appears from accompanying testimony that the spirit used for the preparation of this cordial is derived from a reliable source and well rectified, or, in other words, contains no injurious by-products.

### MARZA WINE.

(Wright, Layman and Umney, Ltd., Southwark, London, S.E.)

Messrs. Wright, Layman and Umney, Ltd., have forwarded to us a bottle of their Marza Wine. This beverage is a tonic wine and contains iron, cocoa, and phosphorus in such quantity as to give in a wineglassful an appreciable dose of these tonics and stimulants. In addition, it contains an active form of pepsin, and hence must be regarded as a digestive. The directions are that the wine should be taken after meals. The iron, however, is present in so bland a form that it might quite well be taken before meals, and is certainly calculated to act as a digestive stimulant. There is abundant scientific testimony as to its usefulness, and we think it is quite worthy to take a prominent place among the standard tonic wines.

